# Workers’ Memorial Day — April 28, 2018

**DOI:** 10.15585/mmwr.mm6716a1

**Published:** 2018-04-27

Workers’ Memorial Day, observed annually on April 28,* recognizes workers who were injured, became ill, or died because of exposures to hazards at work. In 2016, work-related injuries claimed the lives of 5,190 U.S. workers, and the fatal injury rate (3.6 per 100 full time equivalent workers)^†^ rose for the third consecutive year, to the highest rate since 2010. Although deaths resulting from work-related injuries are captured through surveillance, most deaths resulting from work-related illness are not. In 2007, an estimated 53,445 persons died from work-related illness (*1*). In 2016, employers reported approximately 2.9 million nonfatal work-related injuries and illnesses to private industry workers.^§^

Occupational injuries and illnesses also have economic costs. The societal cost of work-related fatalities, injuries, and illnesses was estimated at $250 billion in 2007, based on methods that focus on medical costs and productivity losses (*1*).

New data on fatal falls overboard in the fishing industry, one of the nation’s most hazardous industries, are reported in this issue of *MMWR* (*2*). Since 1991, the CDC-NIOSH Western States Division has studied fishing safety and developed interventions to reduce the incidence of injuries and fatalities among the nation’s fishermen. More information about commercial fishing safety can be found at https://www.cdc.gov/niosh/topics/fishing/.

## Footnotes

* Established in 1970 by the American Federation of Labor and Congress of Industrial Organizations.

† https://www.bls.gov/news.release/pdf/cfoi.pdf.

§ https://www.bls.gov/news.release/pdf/osh.pdf.
