# *Erratum:* Vol. 67, No. 16

**DOI:** 10.15585/mmwr.mm6720a6

**Published:** 2018-05-25

In the announcement “Workers’ Memorial Day — April 28, 2018” on page 465, the second sentence should have read “In 2016, work-related injuries claimed the lives of 5,190 U.S. workers, and the fatal injury rate (3.6 per **100,000** full time equivalent workers)^†^ rose for the third consecutive year, to the highest rate since 2010.” 

The second reference should have read “Case SL, Lincoln JM, Lucas DL. Fatal falls overboard in commercial fishing—United States, **2000**–2016. MMWR Morb Mortal Wkly Rep 2018;67:465–9.”

